# Intratumoral heterogeneity impacts the response to anti-neu antibody therapy

**DOI:** 10.1186/1471-2407-14-647

**Published:** 2014-09-01

**Authors:** Hyunkeun Song, Tae Oh Kim, Sun Young Ma, Jin-Hee Park, Jae-Hyug Choi, Jin-Ho Kim, Mi Seon Kang, Sang Kyun Bae, Ki Hyaung Kim, Tae Hyun Kim, Su-Kil Seo, Il Whan Choi, Geun Am Song, Eric D Mortenson, Yang-Xin Fu, SaeGwang Park

**Affiliations:** Departmentof Microbiology and Immunology, INJE University College of Medicine, 633-165 GaegumDong, Busanjin Gu, Busan, 614-735 Republic of Korea; Department of Internal Medicine, Haeundae Paik Hospital, INJE University College of Medicine, Busan, Republic of Korea; Department of Radiation Oncology, Kosin University College of Medicine, Busan, 602-702 Republic of Korea; Departmentof Pathology, INJE University College of Medicine, Busan, 614-735 Republic of Korea; Department of Nuclear Medicine, INJE University College of Medicine, Busan, 614-735 Republic of Korea; Department of Surgery, INJE University College of Medicine, Busan, 614-735 Republic of Korea; Departmentof Internal Medicine, Pusan National University School of Medicine, Busan, Republic of Korea; Department of Melanoma Medical Oncology-Research, MD Anderson Cancer Center, Houston, TX 77054 USA; Departmentof Pathology and Committee on Immunology, University of Chicago, 924 E. 57thStreet, BSLC R102, Chicago, IL 60637 USA

**Keywords:** Breast cancer, Anti-HER2/neu antibody, Antibody resistance, Spontaneous metastasis, Epithelial mesenchymal transition, Intratumoral heterogeneity, Animal model

## Abstract

**Background:**

Along with de novo resistance, continued exposure to trastuzumab, an anti-human epidermal growth factor receptor 2 (HER2/neu) antibody, can lead to acquired resistance. In this study, we characterize a new anti-HER2/neu antibody resistant and metastatic mouse breast carcinoma cell line, TUBO-P2J. This cell line was developed during *in vivo* experiments using the antibody sensitive and non-metastatic tumor line TUBO. In addition, TUBO-P2J was used to establish an intratumoral HER2 heterogenous animal tumor model to evaluate the therapeutic effects of anti-HER2/neu antibody.

**Methods:**

After establishing the cell line, TUBO-P2J was characterized regarding its susceptibility to anti-neu antibody and chemotherapeutics, as well as its metastatic potential *in vitro* and *in vivo*. In addition, expression profiles of metastasis related genes were also evaluated. A clinically relevant intratumoral HER2 heterogenous tumor model was established by inoculating mice with tumor cells consisting of TUBO and TUBO-P2J at a ratio of 1,000:1 or 10,000:1. Tumor growth and mouse survival were used to evaluate the therapeutic effects of anti-neu antibody.

**Results:**

The TUBO-P2J cell line is a HER2/neu negative and highly metastatic variant of TUBO. This cell line was resistant to anti-neu antibody therapy, and when inoculated subcutaneously, metastasized to the lungs within 14 days. Compared to the parental TUBO cell line, TUBO-P2J displayed an epithelial-mesenchymal transition (EMT) related gene expression profile including: the loss of E-cadherin, and increased Vimentin, Snail, and Twist1 expression. In addition, TUBO-P2J exhibited increased invasion and migration activity, and was resistant to chemotherapy drugs. Finally, mixed tumor implantations experiments revealed that an increased percentage of TUBO-P2J rendered tumors less responsive to anti-neu antibody therapy.

**Conclusion:**

This study describes a novel model of intratumoral heterogenous metastatic breast cancer in immune competent mice that can be used to develop novel or combined immunotherapies to overcome antibody resistance.

**Electronic supplementary material:**

The online version of this article (doi:10.1186/1471-2407-14-647) contains supplementary material, which is available to authorized users.

## Background

Gene amplification and/or overexpression of the human epidermal growth factor receptor 2 (HER2, ErbB-2) have been identified in 20-25% of breast cancers and are associated with poor prognosis [1]. Trastuzumab (herceptin) is a humanized, recombinant monoclonal antibody that binds to the extracellular, juxtamembrane domain of HER2, and is standard care for patients whose breast cancer cells show strong immunohistochemical staining for HER2 or moderate immunohistochemical staining with HER2/neu gene amplification [2]. Trastuzumab therapy has improved response rates, time to relapse and overall survival in woman with HER2-positive metastatic breast cancer [1, 3]. Despite the clinical benefit resulting from trastuzumab administration, primary and acquired clinical resistance has been increasingly reported. To overcome resistance, various efforts including the recognition of primary and acquired drug resistance, studies for the molecular mechanisms of resistance, and developing new drugs and strategies were conducted; however, new approaches are still required.

For some tumors, the epithelial-mesenchymal transition is considered the first step of the metastatic process. Metastatic breast cancers likely evolve from less aggressive epithelial-like breast tumors through reactivation of embryonic signaling pathways and programs like epithelial-mesenchymal transition (EMT) [4]. EMT can be initiated by a diverse set of stimuli including growth factor signaling, tumor-stromal cell interactions and hypoxia [5], but immune responses can also induce EMT through immunoediting [6]. In addition, the EMT does not have to be induced in every cell for metastases to arise. Human primary tumors consist of heterogeneous populations of cells that are phenotypically, functionally and genetically diverse [7]. Even though reports for intratumoral HER2 heterogeneity are increasing, there are no valuable pre-clinical animal models to test whether trastuzumab or HER2 targeted treatments are effective or to develop new treatment strategies.

Recent studies have demonstrated that anti-neu therapy not only directly suppress neu-positive tumors, but also triggers host immune responses for tumor regression [8, 9]. This suggests that strategies aimed at increasing the immune response generated by anti-neu therapy may also limit acquired resistance and reduce metastatic burden. Thus, developing models to study immune activation in the context of neu resistant tumors is of great importance. In this study, we employ a novel metastatic breast cancer tumor model, TUBO-P2J, to discover mechanisms promoting metastatic progression in breast cancer, and susceptibility to chemotherapeutics and anti-neu therapy. Furthermore, using this cell line we further explored how intatumoral HER2 heterogenity affected resistance to anti-HER2/neu therapy.

## Methods

### Mice

Female BALB/c mice were purchased from Orient bio (Taejun, Korea). All mice used in this study were 6–16 weeks of age in accordance to the animal experimental guidelines set by the Institutional Animal Care and Use Committee (IACUC) at the INJE University College of Medicine. This study was approved by the INJE University College of Medicine IACUC (protocol Number 2011-043).

### Cell line and reagents

TUBO was cloned from a spontaneous mammary tumor in a BALB Neu Tg mouse [10]. TUBO cells were cultured in 5% CO_2_, and maintained *in vitro* in DMEM supplemented with 10% heat-inactivated fetal bovine serum (FBS) (Sigma), 10% NCTC 109 medium, 2 mmol/L L-glutamine, 0.1 mmol/L MEM nonessential amino acids, 100 units/mL penicillin, and 100 μg/mL streptomycin. The anti-neu monoclonal antibody 7.16.4 was produced in house. MMP9 specific inhibitor (CAS 1177749-58-4, IC_50_ for MMP9 = 5 nM, IC_50_ for MMP1 = 1.05 μM) was purchased from SantaCruz.

### Isolation of metastatic tumor cells

Metastatic TUBO variant cell (TUBO-P2J) was isolated from metastatic lung nodules by digestion with 1.5 mg/mL collagenase and 100 ug/mL DNase for 20 minutes at 37°C and then gently pipetted in the presence of 0.01 M EDTA (ethylene diaminetetraacetic acid) for 1 minute. Single-cell suspensions were cultured with the same media used for TUBO cells supplemented with G418 (500 μg/ml).

### Migration and invasion assays

The migration potential of TUBO and TUBO-P2J was evaluated with scratch wound and trans-well migration assays. Invasion assays were conducted with matrigel coated trans-well plates. For scratch wound assays, tumor cells were inoculated into a 6-well plate and incubated until cells were approximately 80% confluent. “Wounded” monolayers were created by scraping the bottom of the wells with a sterile pipette tip. After washing twice with PBS, cells were incubated for additional 3 days. Cell migration into the “wound” was determined by microscopic observation. Trans-well experiments were performed using 8.0-um pore size 24-well insert systems (BD Falcon) with 2 mg/ml of Matrigel coating (invasion) or not (migration). 5 × 10^4^ cells (migration) or 5 × 10^5^ cells (invasion) were added to the upper chamber and incubated for 4 hours (migration) or 72 hours (invasion). After incubation, the upper surface of the membrane was wiped with a cotton-tipped applicator to remove residual cells. Cells in the bottom compartment were fixed and stained with H&E. Cells in four randomly selected fields at × 400 magnifications were counted.

### Zymography

For analysis of proteolytic capacity, culture supernatants of TUBO and TUBO-P2J cells were concentrated with Aquacide (Sigma) and diluted to a final protein concentration of 1 mg/ml, and then mixed with sample buffer containing sodium dodecyl sulfate (SDS), glycerol, and bromophenol blue. Equal amounts of each sample were separated on an SDS-polyacrylamide gel (7.5%) containing 0.8 mg/ml gelatin (Merck, Darmstadt, Germany). After electrophoresis, the gels were washed twice with 2.5% Triton × 100 for 30 min to remove any remaining SDS, then washed twice with distilled water and were finally equilibrated with incubation buffer (100 mM Tris/HCl, 30 mM CaCl2, 0.01% NaN3). The gel was then incubated in incubation buffer for 20 hours at 37°C. Staining of protein was performed with Coomassie Blue solution (10 ml of acetic acid, 40 ml of distilled water, 50 ml of methanol, 0.25% Coomassie Blue G250 [SERVA, Heidelberg, Germany]) for 40 min. De-staining was performed in methanol/acetic acid/distilled water (25:7:68, by vol.). After staining, white bands on blue gels indicate enzyme species.

### RT-PCR

Total RNA extracted from cultured cells was used as a template for reverse transcriptase reaction. Aliquots of cDNA were amplified using the primers (Table 1). After an initial denaturation at 94°C for 5 min, the following was performed: 30 cycles of denaturation at 94°C for 30 seconds, annealing at 55 -60°C for 30 seconds, and extension at 72°C for 60 seconds. The reaction products were analyzed in 1.5% agarose gels. The amplified DNA fragments were cloned and sequenced in order to confirm the PCR products.

**Table 1:** **Information on primers used in RT-PCRs**

Genes	NCBI No.	Forward (5′-3′)	Reverse (5′-3′)	Size(bp)
MMP1a	NM_032006	AGACTTCTCTGGTTGCCG	AGAGCCTCCAATCACTGTGC	210
MMP2	NM_008610	CTATTCTGTCAGCACTTTGG	CAGACTTTGGTTCTCCAACTT	309
MMP3	NM_010809	TGTACCAGTCTACAAGTCCTCCA	CTGCGAAGATCCACTGAAGAAGTAG	659
MMP7	NM_010810	CTGCCACTGTCCCAGGAAG	GGGAGAGTTTTCCAGTCATGG	175
MMP8	NM_008611	TGACTCTGGTGATTTCTTGCTAA	GTGAAGGTCAGGGGCGATGC	164
MMP9	NM_013599	CTCAGAGATTCTCCGTGTCCTGTA	GACTGCCAGGAAGACCTTGGTTA	241
MMP10	NM_019471	AGTTGCTCCTGCATGTTCTG	TGCATCCTCTCACCTACTGC	120
MMP11	NM_008606	CCGGAGAGTCACCGTCATC	GCAGGACTAG GGACCCAATG	110
MMP14	NM_008608.3	CTGATGACGATCGCCGTGGCATCC	GCGTCTGAAGAAGAAGACAGCGAGG	878
CDH1	NM_009864	CCATTTTCACGCGCGCTG	CGCGAGCTTGAGATGGAT	396
CDH2	NM_007664	AGCGCAGTCTTACCGAAGG	TCGCTGCTTTCATACTGAACTTT	110
Krt18	NM_010664	CAGCCAGCGTCTATGCAGG	CCTTCTCGGTCTGGATTCCAC	123
Claudin1	NM_016674	GGGGACAACATCGTGACCG	AGGAGTCGAAGACTTTGCACT	100
Occludin	NM_008756	TTGAAAGTCCACCTCCTTACAGA	CCGGATAAAAAGAGTACGCTGG	129
Ctnna1	NM_009818	AAGTCTGGAGATTAGGACTCTGG	ACGGCCTCTCTTTTTATTAGACG	115
Ctnnb1	NM_007614	ATGGAGCCGGACAGAAAAGC	CTTGCCACTCAGGGAAGGA	108
Jup	NM_010593	TGGCAACAGACATACACCTACG	GGTGGTAGTCTTCTTGAGTGTG	135
DDR2	NM_022563	ATCACAGCCTCAAGTCAGTGG	TTCAGGTCATCGGGTTGCAC	116
Fibronectin	NM_010233	AGAGCAAGCCTGAGCCTGAAG	TCGCCAATCTTGTAGGACTGACC	192
FOXC2	NM_013519	AACCCAACAGCAAACTTTCCC	GCGTAGCTCGATAGGGCAG	130
S100a4	NM_011311	TGAGCAACTTGGACAGCAACA	TTCCGGGGCTCCTTATCTGGG	124
SNAI1	NM_011427	CACACGCTGCCTTGTGTCT	GGTCAGCAAAAGCACGGTT	133
SNAI2	NM_011415	TGGTCAAGAAACATTTCAACGCC	GGTGAGGATCTCTGGTTTTGGTA	131
Acta2	NM_007392	GTCCCAGACATCAGGGAGTAA	TCGGATACTTCAGCGTCAGGA	102
Twist1	NM_011658	GGACAAGCTGAGCAAGATTCA	CGGAGAAGGCGTAGCTGAG	146
Vimentin	NM_011701	CGGCTGCGAGAGAAATTGC	CCACTTTCCGTTCAAGGTCAAG	124
GAPDH	NM_008084	TTCACCACCATGGAGAAGGC	GGCATGGACTGTGGTCATGA	250

### Real time –PCR

Quantitative real-time reverse transcription-PCR (qRT-PCR) was performed with fluorescent SYBR Green using an ABI Step One Plus system (Applied Biosystems) following the manufacturer’s instructions. The standard glyceraldehydes-3-phosphate dehydrogenase (GAPDH) was used to normalize variations in input cDNA. Quantitative PCR reactions were performed in triplicate.

### Flow cytometry

To determine the surface expression of rat *neu*, cells were harvested and washed with phosphate buffered saline (PBS). The cells were then suspended with 0.5% bovine serum albumin (BSA) in PBS and then each labeled with 1ug/ml of anti-neu antibody. After incubation for 30 min at 4°C, data acquisition and flow cytometry analysis were performed with FACS Calibur using the Cell Quest software (BD Biosciences).

### Western blot

Cell lysate (30ug/lane) was electrophoresed on polyacrylamide-SDS gel and then transferred to polyvinylidene fluoride membrane. Immunoblotting was performed by various primary antibodies. E-cadherin, Vimentin and Snail 1 antibodies were obtained from Cell Signaling Technology (Beverly, MA). Actin antibody was obtained from Santa Cruz Biotechnology (Santa Cruz, CA). Twist antibody was purchased from Abcam (Cambridge, MA). Western blots are representative of three independent experiments.

### *In vitro*proliferation assay

Cell viability was measured using the Cell Proliferation Reagent WST-1 (Roche Diagnostics, IN, USA) according to the manufacturer’s protocol. 1-2 × 10^3^ cells of TUBO or TUBO-P2J cells were seeded in 96-well plates and incubated with various concentrations of antibody or chemo drugs for 72 hours. WST-1 reagent was added and incubated with the cells for 1 hour. The absorbance was determined at 450 nm with an ELISA plate reader.

### Tumor inoculation and antibody treatments

TUBO or TUBO-P2J cells were detached from culture flasks by incubating for 3–5 minutes in 1 × Trypsin EDTA (Mediatech Inc., Manassas, VA). Cells were washed 2–3 times in 1× PBS and counted by trypan blue exclusion. 2-5 × 10^5^ TUBO, TUBO-P2J or a mixture of the two cells was injected subcutaneously in the back of 6 to 8-week-old mice anesthetized with a mixture of ketamine (90 mg/kg) and xylazine (10 mg/kg). Tumor volumes were measured along three orthogonal axes (x, y, and z) and calculated as tumor volume = (xyz)/2.

Tumor bearing mice were treated with 2 or 4 times with intraperitoneal injections of 100 to 200 μg of anti-neu antibody (clone 7.16.4).

### Immunohistochemistry

Tumor tissues were fixed in 4% paraformaldehyde and then were embedded in paraffin blocks. Tissue sections from a paraffin block (4 μm thick) were incubated in tris-EDTA buffer (pH 8.0) and heated to 99°C for 30 min. After the endogenous peroxidase activity was quenched with 3% hydrogen peroxide, the sections were treated with UV inhibitor (Ventana, CA, USA). The sections were incubated with rabbit anti-rNeu antibody (Cell Signaling) and HRP-conjugated goat anti-rabbit IgG (Jackson ImmunoResearch Lab. PA, USA). Finally, counterstaining was performed with Mayer’s hematoxylin. IHC grading was done as following ASCO Clinical Practice Guideline [2].

### Statistical analysis

Differences between groups were analyzed using an unpaired *t* test. Error bars represent ± SD. All statistical analyses were conducted using Graph-Pad Prism Version 4.0 (GraphPad Software). Unless specified, statistically significant differences of P <0.05, 0.01, and 0.001 are noted with *, **, ***, respectively. Differences that were not statistically significant were left unnoted.

## Results

### TUBO-P2J cell line is a HER2/neu loss variant resistant to anti-neu antibody and chemotherateutics

**Figure 1:**
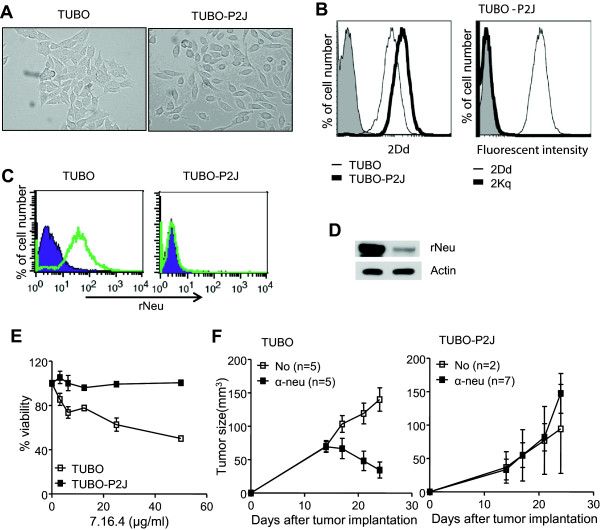
**Expression pattern of surface antigen and differential susceptibility to anti-neu antibody in TUBO and TUBO-P2J cell line. A)** Microscopic images of TUBO and TUBO-P2J cells. **B)** Differential expression of MHC class I molecule, H-2Dd expression of TUBO and TUBO-P2J cells analyzed by flow cytometry is shown (left histogram). Representative analysis of H-2Kq and H-2Kd expression intensity in TUBO-P2J cells is shown (right histogram). The isotype control is represented by the gray shaded region and MHC class I molecule expression is represented by the black line. Data shown in each panel are representative of three independent experiments. **C, D)** Rat Neu protein expression of TUBO and TUBO-P2J cells were evaluated with flow cytometry for surface expression **(C)** and western blot for total protein level **(D)** using purified ascites of 7.16.4 and PE- or HRP-conjugated anti-mIgG. **E)** Inhibition of cell viability by treatment with anti-neu antibody (left graph). Cells were treated with anti-neu antibody (clone 7.16.4) and then cell viability was measured using the Premix WST-1 cell proliferation assay system. The assay was performed in quadruplicate with essentially similar results. Cell viability was recorded as the optical density at 440 to 600 nm relative to that of the sample not treated (mean ± SD). **F)** Therapeutic effects of anti-neu antibody on TUBO (left) or TUBO-P2J (right) bearing mice. WT BALB/c mice (n = 4-6/group) were inoculated s.c. with 4 × 10^5^ TUBO cells or 2 × 10^5^ TUBO-P2J cells and treated i.p. with 200 μg anti-neu (α-neu) antibody or isotype control (Ctrl) on day 12 and 100 μg antibody at 7 days after first treat. The growth of tumor measured once or twice a week. Data shown in each panel are representative of two independent experiments.

Spontaneous metastases have not been reported in previous studies using subcutaneous implantation of TUBO. However, during TUBO transplant experiments in NeuTg F_1_mice (FVB/N-Tg/MMTV-neu × BALB/c), a few anti-neu antibody treated mice became very sick with cachexia. Autopsies were conducted to evaluate the cause of the sickness and revealed that the lymph nodes and spleens of these mice were enlarged and a numbers of nodules were detected in the lungs. The lung nodules were digested with collagenase and implanted subcutaneously on back of wild type BALB/c mice. After 30 days, metastatic tumor nodules were observed in the lungs and subsequently isolated and cloned. The resulting metastatic cell line was named TUBO-P2J. TUBO-P2J and TUBO cell lines displayed a different morphology. As shown in Figure 1A, isolated TUBO-P2J cells exhibited a more fibroblastic-like phenotype than TUBO cells. To rule out the possibility that TUBO-P2J originated from FVB/N-Tg/MMTV-neu, MHC class I was expression was examined. TUBO-P2J cells only expressed H-2K^d^ and not H-2K^q^ (Figure 1B). These data suggest that TUBO-P2J is a metastatic version of the TUBO cell line.

TUBO is a neu-dependent cell line that it is inhibited *in vivo* by anti-neu antibody therapy [8]. Because TUBO-P2J arose after anti-neu antibody treatment, we examined the level of neu expression. Although TUBO-P2J was derived from TUBO, surface expression of the neu protein was not observed by flow cytometry (Figure 1C), and Western blot analysis revealed that total neu protein levels were markedly reduced (Figure 1D). To further evaluate if this alteration affected the susceptibility of TUBO-P2J to antibody therapy, we tested if anti-neu antibody was able to inhibit the growth of TUBO-P2J *in vitro* and *in vivo*. As expected, anti-neu therapy inhibited the growth of TUBO both *in vitro* and *in vivo*. However, the ability of anti-neu therapy to inhibit TUBO-P2J *in vitro* was significantly decreased (Figure 1E). In addition, anti-neu therapy was unable to inhibit the growth of TUBO-P2J tumors in WT mice (Figure 1F). These data suggest that TUBO-P2J is an anti-neu resistant cell line that has metastatic potential.

We also evaluated the susceptibility of TUBO and TUBO-P2J to 12 chemotherapeutics that are commonly used to treat breast cancer patients (Table 2). The IC50 for cisplatin, etoposide and irinotecan in TUBO-P2J were similar or lower than that of TUBO. However, the IC50 for the other 9 drugs was at least 5 times greater. These data suggest that TUBO-P2J is also more resistant to chemotherapy than the parental TUBO cell line.

**Table 2:** **Chemo susceptibility of TUBO and TUBO-P2J cells**

Chemoreagent	IC50	
	TUBO	TUBO-P2J
Carboplatin	0.22 mg/ml	4.97 mg/ml
Cisplatin	4.2 ug/ml	3.53 ug/ml
Cyclophosphamide	0.15 mg/ml	1.93 mg/ml
Daunorubicin	UD	326 ng/ml
Doxoruibicin	56.9 ng/ml	306.6 ng/ml
Epirubicin	2.87 ng/ml	40.3 ng/ml
Etoposide	84 ug/ml	76.63 ug/ml
Gemcitabin	0.55 ng/ml	6.4 ng/ml
Idarubicin	UD	0.98 ug/ml
Irinotecan	1.7 mg/ml	0.95 mg/ml
Oxaliplatin	0.26 ug/ml	34.55 ug/ml
Vinorelbine	0.23 ug/ml	6.8 ug/ml

*UD* under detected.

### TUBO-P2J spontaneously metastasizes to the lung and displays increased migration and invasion activity

**Figure 2:**
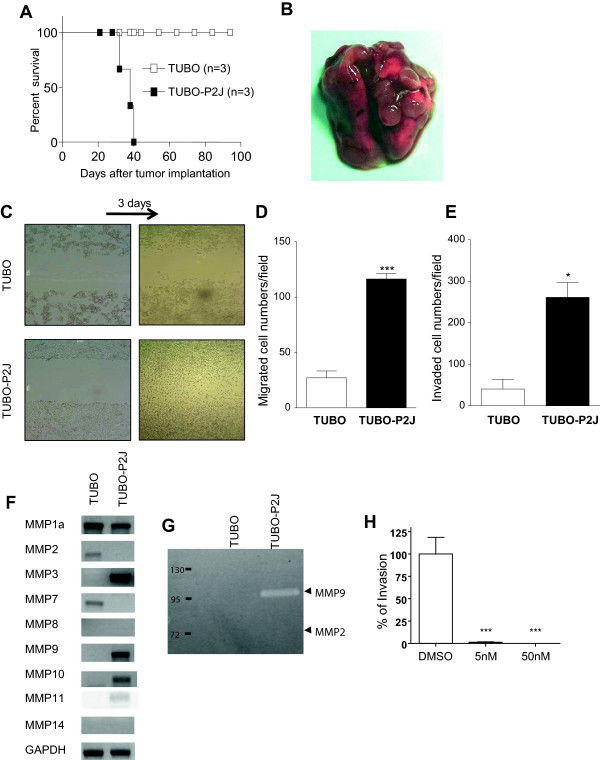
**Metastatic capacity of TUBO-P2J cells** ***in vitro*** **and** ***in vivo*** **. A)** Percent survival of TUBO and TUBO-P2J bearing mice. TUBO (4 × 10^5^ /mice) and TUBO-P2J (2 × 10^5^ /mice) cells were implanted subcutaneously on the backs of mice. Fourteen days after implantation, primary tumors were surgically removed and mouse survival was recorded. **B)** Picture of lungs from TUBO-P2J (2 × 10^4^ /mice) tumor bearing mouse 30 days after implantation. **C)** Scratch wound assay: 1 × 10^6^ cells of TUBO or TUBO-P2J were seeded in 6 well culture plates. The surface of the culture plates was scratched mechanically with pipette tips and cell migration was evaluated 3 days later. **D)** Trans-well migration assay: 5 × 10^4^ cells of TUBO and TUBO-P2J cells were added to the top plate and incubated for 4 hours. Migrated cells were counted from 5 randomly selected fields under × 40 magnification and averages were calculated. Data shown are representative of three independent experiments (mean ± SD). **E)** Invasion assay: 1 × 10^5^cells of TUBO and TUBO-P2J cells were added to matrigel coated trans-well plates and incubated for 3 days. Invaded cells were counted from 5 randomly selected fields under × 40 magnification and averages were calculated. Data shown are representative of three independent experiments (mean ± SD). **F)** RT-PCR for expression of MMPs. **G)** Zymography for MMPs **H)** Inhibition of invasion by MMP9 inhibitor I. Invasion assay was done with indicated doses of MMP9 inhibitor by the same procedure as (E). Data shown are representative of two independent experiments (mean ± SD). * is *p* < 0.05 and *** is *p* < 0.001 versus TUBO or DMSO.

To determine the metastatic capacity of TUBO-P2J, either TUBO or TUBO-P2J cells were injected subcutaneously into WT mice, and on day 14 the established tumors were surgically resected. By day 40, all mice that had been inoculated with TUBO-P2J died; however, mice that had been inoculated with TUBO bearing survived for 100 days (Figure 2A). These data suggest that TUBO-P2J metastases occur by day 14. To confirm this, we used small animal PET-CT to detect metastatic lung masses. Mice inoculated with TUBO-P2J had detectable lung nodules around day 28 after implantation (Additional file 1: Figure S1). In addition, multiple lung nodules were visually evident in mice inoculated with TUBO-P2J around day 30 (Figure 2B). Thus, these data suggest that TUBO-P2J cells maintain their metastatic capacity *in vivo*.

In order to study the intrinsic characteristics driving metastasis of TUBO-P2J, we first evaluated the migration activity of TUBO and TUBO-P2J using scratch wound assay and trans-well migration experiments. First, 1x10^6^ TUBO and TUBO-P2J cells were seeded separately in 6-well plates. Twelve hours after plating, the bottom of each well was scratched with a pipette tip to remove cells and create a “wound”. Within three days, TUBO-P2J cells had migrated and covered much of the exposed plate whereas very little migration was observed in the wells containing TUBO cells (Figure 2C). To further this analysis, we conducted trans-well migration experiments. To this end, 1 × 10^5^ TUBO or TUBO-P2J cells were seeded in the upper chamber, and cell numbers in lower chamber were evaluated 4 hours later. The migration capacity of TUBO-P2J was almost 20 times greater than that of TUBO (P < 0.001) (Figure 2D).

Next, in an effort to determine if these differences promoted the metastatic potential of TUBO-P2J cells, the invasion ability of TUBO and TUBO-P2J cells was compared with a matrigel coated trans-well insert. After a three-day incubation period, a significantly greater number of TUBO-P2J had invaded the matrigel compared to the parental TUBO cells (P < 0.05) (Figure 2E). Expression and enzyme activity of various matrix metalloproteases (MMPs) is associated with cell invasion. RT-PCR analysis revealed that mRNA for MMP3, 9, 10 and 11 were increased in TUBO-P2J (Figure 2F). In addition, gelatin zymopgraphy revealed that MMP9 present in TUBO-P2J cells exhibited increased enzymatic activity (Figure 2G). To test whether the active MMP9 expression caused increase of migration and invasion, MMP9 inhibitor I (CAS 1177749-58-4) was used. Invasion of TUBO-P2J cells was reduced to more than 95% by 5 nM of MMP9 inhibitor I (IC_50_ for MMP9) and blocked completely by 50 nM (Figure 2H). However, migration of TUBO-P2J cells was not changed by MMP9 inhibitor (Additional file 2: Figure S2). Collectively, these data demonstrate that TUBO-P2J has acquired increased invasive capabilities over TUBO, and that these characteristics may be due to increased MMP9 activity.

### TUBO-P2J displays characteristics of a transition from epithelial to mesenchymal (EMT) cells

**Figure 3:**
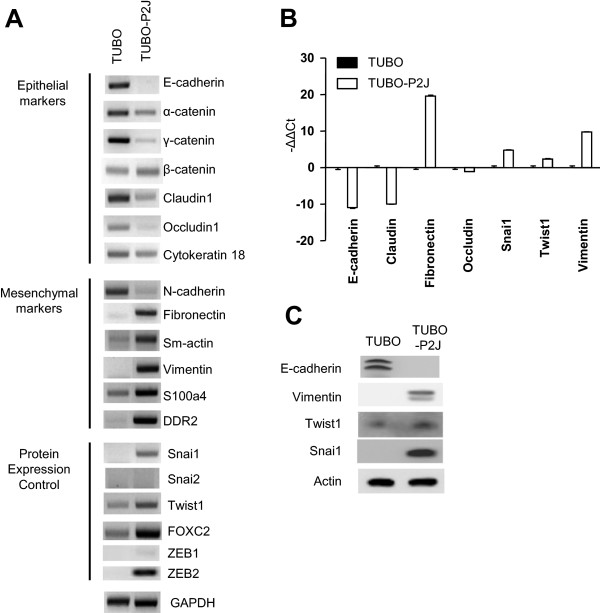
**Analysis of EMT profiles in TUBO-P2J cells. A)** Measurement of mRNA expression of epithelial and mesenchymal markers and EMT gene expression regulators by RT-PCR. **B)** Quantitative real-time PCR of EMT related genes(mean ± SD). **C)** EMT related protein expression of TUBO and TUBO-P2J cells.

To determine the potential molecular changes driving metastatic transition of TUBO cells, we conducted a cDNA array between TUBO and TUBO-P2J (Additional file 3: Figure S3). The expression of hundreds of genes was increased or decreased in TUBO-P2J cells compared to the parental TUBO cells. As a result, the gene expression profile of TUBO-P2J resembled that of the spontaneous metastatic breast carcinoma cell line 4T1. Among the differences, the most prominent changes in TUBO-P2J were loss of E-cadherin expression and gain of Fibronectin and Vimentin expression. Generally, loss of E-cadherin is a hallmark of epithelial-mesenchymal transition (EMT). To confirm whether the metastatic capability of TUBO-P2J cells was associated with acquisition of mesenchymal characteristics, expression of known EMT genes was evaluated with RT-PCR (Figure 3A). E-cadherin was expressed in TUBO cells, but absent in TUBO-P2J. Other epithelial makers such as α-catenin, γ-catenin, Claudin1, Occludin1, and Cytokeratin18 were expressed in TUBO-P2J, but at significantly decreased levels from those observed in the parental TUBO line. In contrast, the mesenchymal makers (Fibronectin, Sm-actin, Vimentin, S100A4, and DDR2) and transcription regulators (Snail1, Twist1, and FOXC2) for mesenchymal cells were significantly increased in TUBO-P2J (Figure 3A). Additionally, quantitative PCR (qRT-PCR) of TUBO or TUBO-P2J cells revealed that TUBO-P2J cells displayed characteristics of mesenchymal cells while TUBO cells maintained more epithelial characteristics (Figure 3B). To determine if these differences were also evident at the protein level, we conducted Western blot analysis. Compared to TUBO cells, E-cadherin expression was reduced in TUBO-P2J cells. However, mesenchymal cell proteins such as vimentin, Snail, and Twist1, were markedly enhanced in TUBO-P2J cells. These data suggest that the EMT process might be related in phenotype changes of TUBO cells to TUBO-P2J cells.

### Intratumoral HER2 heterogeneity impacts the efficacy of anti-HER2/neu therapy

**Figure 4:**
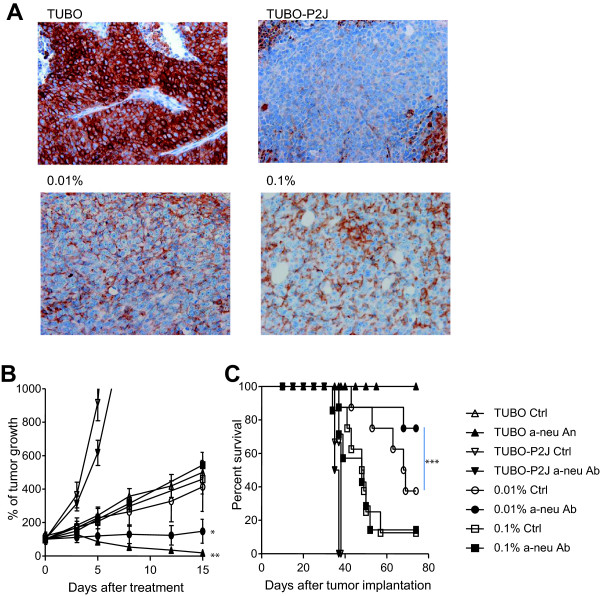
**Tumor heterogeneity impacts response to anti-neu antibody therapy.** WT BALB/c mice (n = 6-8/group) were inoculated s.c. with a mixture of 5 × 10^5^ TUBO and TUBO-P2J cells. The percentage of TUBO-P2J cells was either 0.1% or 0.01%. **A)** Immunochemistry staining of mixed tumors for Neu. **B-C)** Mice were treated i.p. with 200 μg anti-neu (α-neu) antibody or isotype control (Ctrl) on day 10, and 100 μg on day 13, 16, and 19. Tumors were resected on day 21. The therapeutic effects of anti-neu antibody were evaluated by measuring primary tumor growth **(B)** and overall survival **(C)**. The sizes of tumor were measured twice a week. *P < 0.05, ** *P* <0.01 and ***P < 0.001 versus control group. One of two experiments is shown.

Breast cancer patients express different levels of surface HER2/neu, and are characterized as being either +1 to +3 by immunohistochemistry (IHC) [2]. However, even in +3 tumors not every cell positive for HER2/neu [11]. Thus, there is intratumoral heterogeneity within human breast cancer tumors, and this may often include highly metastatic cells. Considering this fact, we took advantage TUBO-P2J to develop a heterogeneic tumor model. To this end, we mixed TUBO cells with TUBO-P2J cells at various ratios (1,000:1 or 10,000:1) prior to inoculation. We then evaluated whether these mixed tumors showed intratumoral heterogeneity and how these heterogeneic tumors responded to anti-neu antibody treatment in a neo-adjuvant setting (Figure 4). After transplanted tumors were established (50-100 mm^3^), HER2/neu status of mixed tumor was evaluated with IHC using rabbit anti-rNeu antibody and HRP-conjugated anti-rabbit IgG. It is hard to grade of IHC results because antigen and antibody are not the same with that in human. But we grade IHC results based on guideline of HER2 testing in human breast cancer [2]. IHC results showed that TUBO tumor is HER2/neu positive (IHC 3+) because all the tumor cells were stained completely on membrane and TUBO-P2J tumor is HER2/neu negative (IHC 0-1+) because most cells were not stained and even though some cells were stained but it was vary week and faint. Mixed tumors of both ratios are IHC 2+ or equivocal. More than 30% of tumor cells were positive in both mixed tumors, but the staining intensity was moderate than TUBO tumors. The percentages of positive cells were slightly higher in 0.1% mixed tumor than 0.01%.Tumor-bearing mice were treated with anti-neu antibody intraperitoneally 4 times at 3-day intervals. Three days after the last anti-neu treatment, the primary tumors were surgically removed. Prior to anti-neu therapy, the growth of heterogeneic tumors was similar to that of the homogenous TUBO inoculations; however, the efficacy of anti-neu antibody therapy was diminished with addition and increasing ratio of TUBO-P2J (Figure 4B & C). Even though mixed tumors were not regressed like as TUBO homogenous tumor, but anti-neu antibody therapy significantly delayed tumor growth (p = 0.014) and increased mouse survival (p < 0.001) when the heterogeneic ratio was 10,000:1 (0.001%). This effect was abolished, however, when the ratio was 1,000:1 (0.01%). At this ratio anti-neu antibody no longer suppressed tumor growth and mouse survival was reduced. These data support clinical observations that the percentage of HER2/neu + within the tumor and IHC grades of tumors correlate with response to anti-HER2/neu therapy, and demonstrates that our heterogenous tumor model is useful for future studies aimed at developing combination therapies for low or equivocal HER2/neu + tumors.

## Discussion

Even though trastuzumab provides clinical benefit to breast cancer patients, resistance to trastuzumab develops. About 70% of patients who initially respond to trastuzumab eventually experience progression to metastatic disease within a year [12] and only 11-26% of Her2+ metastatic breast cancers respond to trastuzumab monotherapy [13]. Therefore, understanding the mechanisms for trastuzumab resistance and developing treatment strategies for primary and acquired resistance are important. In this study, we sought to define the molecular and functional changes driving loss of HER2/neu expression and metastatic potential by examining a spontaneously derived metastatic breast cancer cell line, TUBO-P2J, with the parental HER2/neu positive cell line, TUBO. In addition, we used this metastatic variant to establish a clinically relevant intratumoral HER2 heterogeneous tumor model to test anti-HER2/neu antibody susceptibility. Using this model, we observed that the clinical benefit of anti-neu antibody monotherapy correlated with heterogeneity of HER2/neu.

TUBO is rNeu positive, and highly susceptible to anti-HER2/neu antibody therapy (clone 7.16.4) *in vitro* and *in vivo* [8]. Unlike TUBO, rNeu expression inTUBO-P2J is significantly reduced and cell surface levels were undetectable by flow cytometry. The loss of rNeu expression also rendered TUBO-P2J unresponsive to anti-neu antibody *in vitro* and *in vivo*. These results were similar to previous reports that neu-specific immunotherapy, including anti-neu antibody and vaccines, induced antigen loss variants in FVB/N-TgN and FVB/N WT mice through immunoediting [14, 15]. Antigen loss has also been observed in patients treated with the therapeutic antibodies, trastuzumab and rituximab [16, 17]. Taking these reports and our results together suggest that immunoediting might be a one of mechanisms promoting resistance to antibody-based treatments.

The metastatic process consists of a series of steps including: migration, invasion, intravasation, arrest, extravasation, and colonization; all of which must be successfully completed in order to give rise to a metastatic tumor [18–20]. TUBO cells are only capable of colonizing the lung after intravenous injection [21]. TUBO-P2J cells, however, successfully established lung metastatic nodules when implanted subcutaneously on the back of mice, and these metastases arose within 14 days after implantation. These data suggest that TUBO cells have a limitation in the metastasis steps before intravasation, perhaps migration or invasion. Our data revealed thatTUBO-P2J has significantly increased migration and invasion activity over the parental TUBO cell line. Knutson et al [14, 15] suggested that immunoediting could induce antigen negative variants through epithelial-mesenchymal transition (EMT). The EMT process is characterized by the loss of epithelial markers and gain of mesenchymal markers [22]. Generally, loss of E-cadherin is a hallmark of EMT. TUBO cells expressed E-cadherin, but TUBO-P2J cells did not. Except for the loss of N-cadherin expression, the gene expression profile of TUBO-P2J represented EMT. Most notably was the increased expression of the mesenchymal cell markers DDR2, Snail 1, fibronectin, Sm-actin, Twist, and FOXC2, and the reduced expression of the epithelial cell markers Cytokeratin, Claudin, and Occludin. Thus, TUBO-P2J might be another example for EMT and anti-HER2/neu resistance induced by immunoediting. EMT also correlates with drug resistance [23]. Mice inoculated with TUBO-P2J were less sensitive to both anti-neu therapy and chemotherapeutic drugs. The IC_50_ for 9 of 12 chemo drugs was increased 5-130 times for TUBO-P2J (Table 2).

Intratumoral HER2 heterogeneity also exists [1, 11, 24]. Thus, one of clinical feature of resistance to trastuzumab might be intratumoral HER2 heterogeneity. Using TUBO-P2J, we developed a heterogenous tumor model through implantation of mixtures of TUBO and TUBO-P2J. We expected that HER2/neu status should be 3+ when the ratio of TUBO cells in mixture was higher than 99%. However the IHC status of both 0.1% and 0.01% mixed tumor is 2+ or equivocal. The percentages of HER2/neu positive cells were lower than expected. In addition, intensity of HER2/neu positive cells in mixed tumors was weak than that of TUBO tumor. Increased percentages of HER2/neu negative cells than expected can be explained with differences of cell proliferation rates in *in vitro* and *in vivo*. However, it is hard to understand why the intensity of HER2/neu positive cells in mixed tumors is weak. It is not clear but might be caused by cellular interaction between TUBO and TUBO-P2J.

In previous studies, we reported that anti-neu antibody can induce anti-tumor CD8+ and CD4+ T cell responses [8, 25]. Because TUBO and TUBO-P2J share many antigens, we hypothesized that if anti-neu antibody was able to target a majority of the tumor cells, it would still reduce tumor burden. Mice inoculated with tumors consisting of 0.01% TUBO-P2J responded to anti-neu therapy and displayed increased survival. However, when the percentage of TUBO-P2J was increased to 0.1%, anti-neu therapy no longer provided benefit on tumor mass and survival. In these experiments, tumor bearing mice were treated in a neo-adjuvant setting, which raises the possibility that a single targeting treatment can select non-targeted cells and can lose efficacy over time.

In conclusion, we isolated a spontaneous metastatic breast cancer cell line, TUBO-P2J. This cell line displayed markers of EMT, and exhibited an increased metastatic capacity compare to parental TUBO cells. Furthermore, using this cell line we developed a mixed tumor model to mimic clinical tumor heterogeneity and low HER2/neu + tumor. Overall, this study emphasizes the importance of combination therapy targeting multiple antigens or oncogenic mechanisms, and provides a means to test these therapies with more clinical relevancy.

## Conclusion

This study describes a novel model of intratumoral heterogenous metastatic breast cancer in immune competent mice that can be used to develop novel or combined immunotherapies to overcome antibody resistance.

## Electronic supplementary material

Additional file 1: Figure S1: PET-CT imaging of TUBO-P2J bearing mice. 2 × 10^4^ cells of TUBO-P2J were injected subcutaneously into the lower back of mice. Lung metastases were evaluated with small animal PET-CT after intra-peritoneal injection of [18 F]. Images represent PET-CT at days 7 days, 14 and 28. (PPTX 917 KB)

Additional file 2: Figure S2: Migration and Invasion assay with MMP9 inhibitor. 5 × 10^4^ (for migration) or 1 × 10^5^ (for invasion) of TUBO-P2J cells were added to the top plate with or without matrigel coating and incubated for 4 hours (for migration) or 3 days (for invasion). Cells in the bottom compartment were fixed and stained with H&E. (PPTX 395 KB)

Additional file 3: Figure S3: Hierarchical clustering for the differentially expressed genes between TUBO, TUBO-P2J, and 4T1 cell lines. Relative gene expression levels of TUBO-P2J and 4T1 compare to that of TUBO cell are showed as colored bars representing expression levels for a given gene from cDNA array, aligned in a row and each cell is a different column. Red indicates increased expression, black is unchanged and green is reduced, all relative to a control sample. (PPTX 77 KB)

## Abbreviations

HER2 (Erbb-2): Human epidermal growth factor receptor 2
EMT: Epithelial-mesenchymal transition
MMPs: Matrix metalloproteases
qRT-PCR: quantitative real time -PCR.
