# Supplementary figures and images for: Intratumoral heterogeneity impacts the response to anti-neu antibody therapy
Source: BMC Cancer. 2014 Sep 1;14:647. doi: 10.1186/1471-2407-14-647 (PMC4161915; doi:10.1186/1471-2407-14-647)

## Slide 1
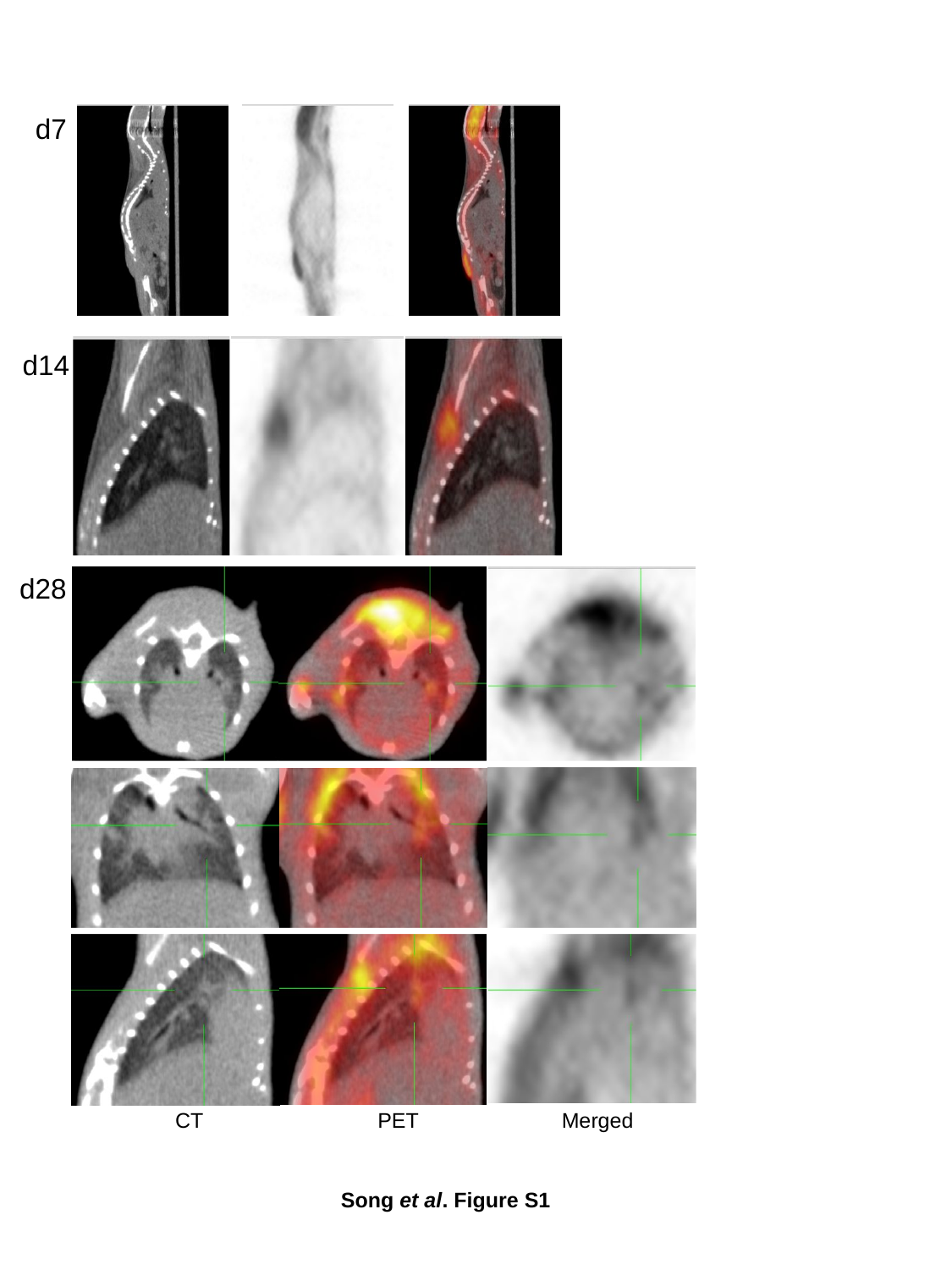

d7
d14
d28
CT
PET
Merged
Song et al. Figure S1

Supplement: Supplementary file 1 — Additional file 1: Figure S1: PET-CT imaging of TUBO-P2J bearing mice. 2 × 10^4^ cells of TUBO-P2J were injected subcutaneously into the lower back of mice. Lung metastases were evaluated with small animal PET-CT after intra-peritoneal injection of [18 F]. Images represent PET-CT at days 7 days, 14 and 28. (PPTX 917 KB) [file 12885_2013_4825_MOESM1_ESM.pptx]

## Slide 1
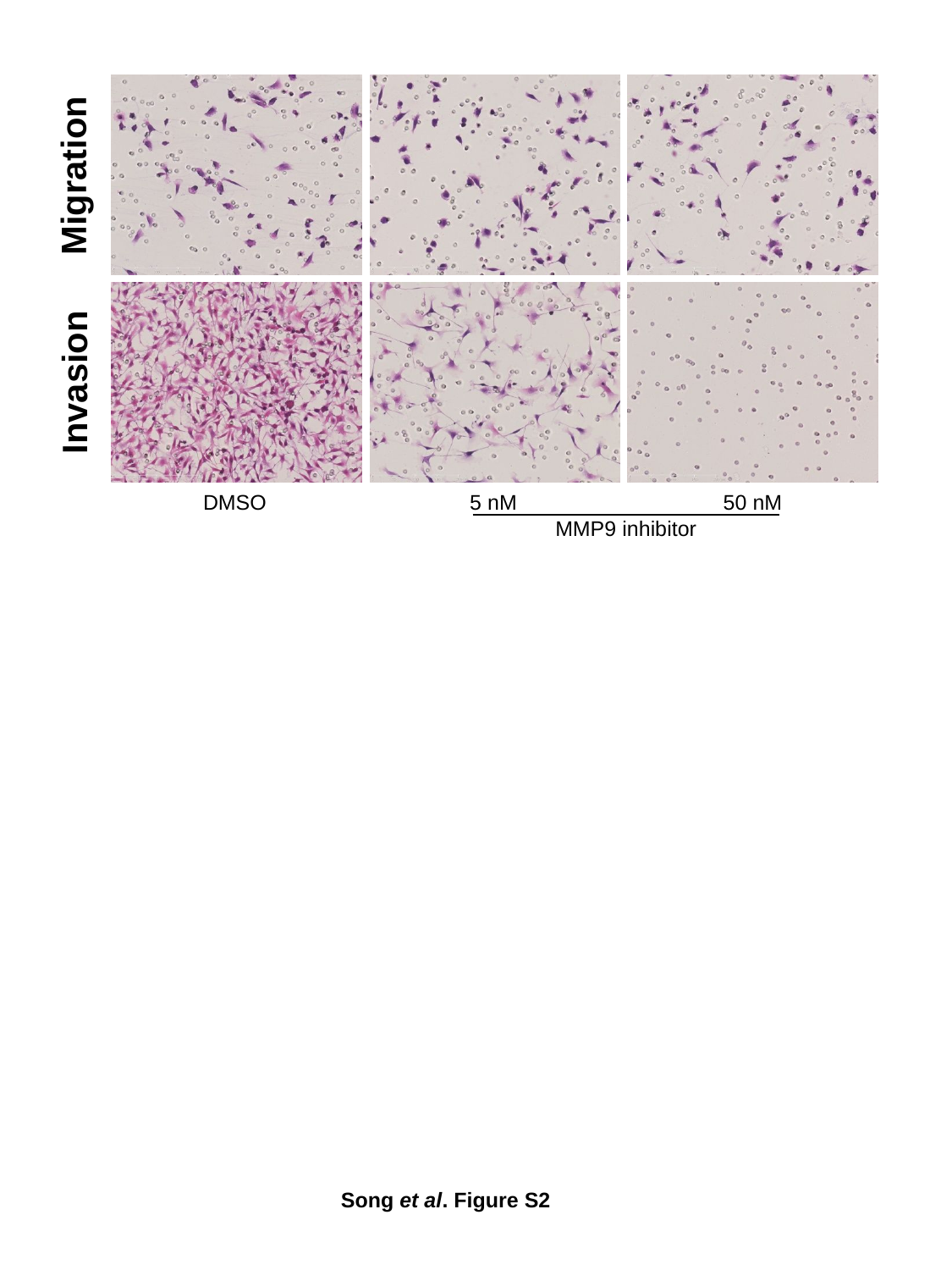

Migration
Invasion
DMSO
5 nM
50 nM
MMP9 inhibitor
Song et al. Figure S2

Supplement: Supplementary file 2 — Additional file 2: Figure S2: Migration and Invasion assay with MMP9 inhibitor. 5 × 10^4^ (for migration) or 1 × 10^5^ (for invasion) of TUBO-P2J cells were added to the top plate with or without matrigel coating and incubated for 4 hours (for migration) or 3 days (for invasion). Cells in the bottom compartment were fixed and stained with H&E. (PPTX 395 KB) [file 12885_2013_4825_MOESM2_ESM.pptx]
