# Supplementary material for: Intratumoral heterogeneity impacts the response to anti-neu antibody therapy
Source: BMC Cancer. 2014 Sep 1;14:647. doi: 10.1186/1471-2407-14-647 (PMC4161915; doi:10.1186/1471-2407-14-647)
Supplement: Supplementary file 3 — Additional file 3: Figure S3: Hierarchical clustering for the differentially expressed genes between TUBO, TUBO-P2J, and 4T1 cell lines. Relative gene expression levels of TUBO-P2J and 4T1 compare to that of TUBO cell are showed as colored bars representing expression levels for a given gene from cDNA array, aligned in a row and each cell is a different column. Red indicates increased expression, black is unchanged and green is reduced, all relative to a control sample. (PPTX 77 KB) [file 12885_2013_4825_MOESM3_ESM.pptx]

## Slide 1
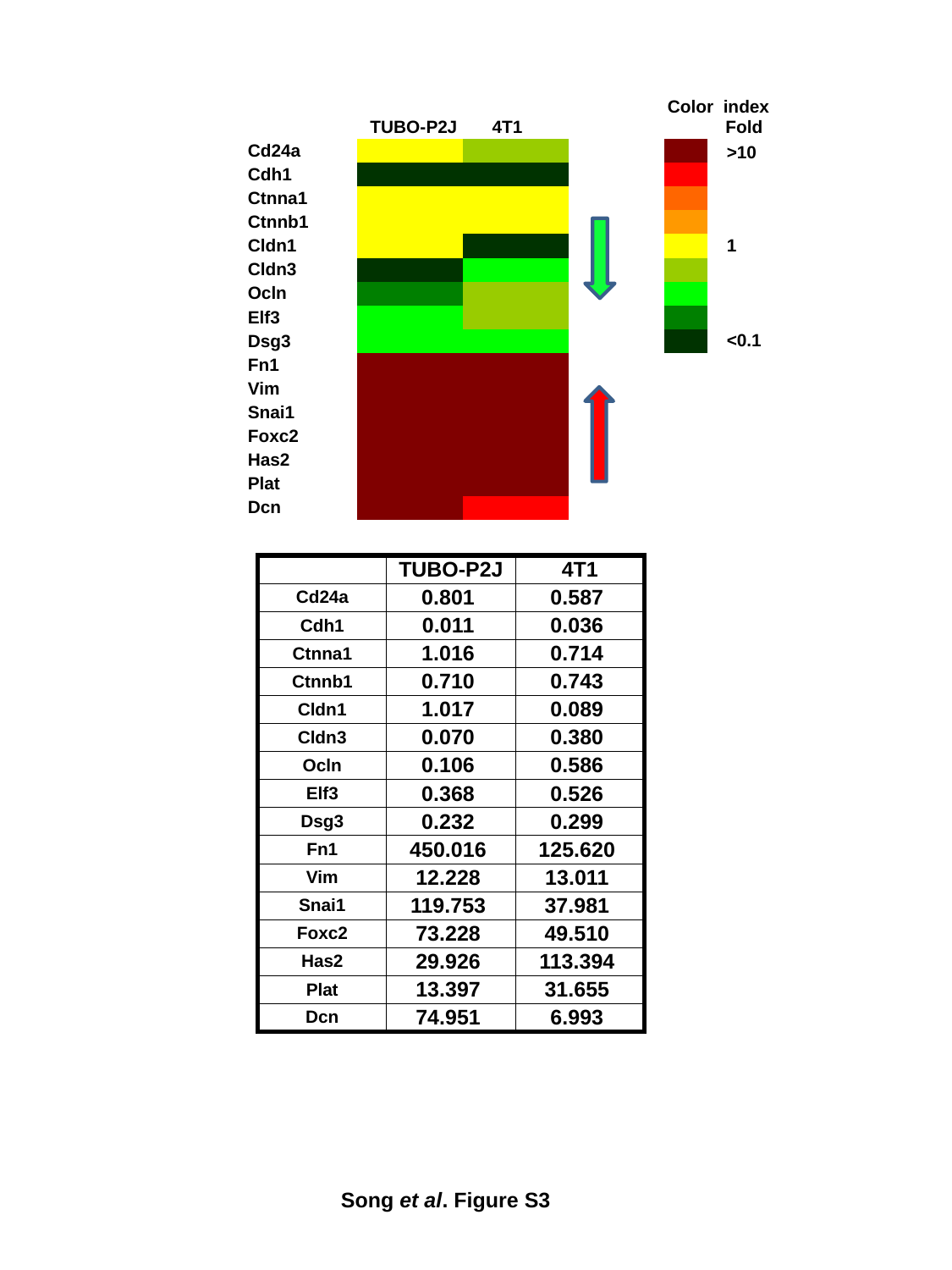

Color index
TUBO-P2J
4T1
Fold
>10
| Cd24a |
| --- |
| Cdh1 |
| Ctnna1 |
| Ctnnb1 |
| Cldn1 |
| Cldn3 |
| Ocln |
| Elf3 |
| Dsg3 |
| Fn1 |
| Vim |
| Snai1 |
| Foxc2 |
| Has2 |
| Plat |
| Dcn |
| | |
| --- | --- |
| | |
| | |
| | |
| | |
| | |
| | |
| | |
| | |
| | |
| | |
| | |
| | |
| | |
| | |
| | |
| |
| --- |
| |
| |
| |
| |
| |
| |
| |
| |
1
<0.1
| | TUBO-P2J | 4T1 |
| --- | --- | --- |
| Cd24a | 0.801 | 0.587 |
| Cdh1 | 0.011 | 0.036 |
| Ctnna1 | 1.016 | 0.714 |
| Ctnnb1 | 0.710 | 0.743 |
| Cldn1 | 1.017 | 0.089 |
| Cldn3 | 0.070 | 0.380 |
| Ocln | 0.106 | 0.586 |
| Elf3 | 0.368 | 0.526 |
| Dsg3 | 0.232 | 0.299 |
| Fn1 | 450.016 | 125.620 |
| Vim | 12.228 | 13.011 |
| Snai1 | 119.753 | 37.981 |
| Foxc2 | 73.228 | 49.510 |
| Has2 | 29.926 | 113.394 |
| Plat | 13.397 | 31.655 |
| Dcn | 74.951 | 6.993 |
Song et al. Figure S3
